# Paired dosimetric comparison of VMAT-based total body and total marrow irradiation in adult leukemia patients: enhanced organ sparing with consistent plan complexity

**DOI:** 10.3389/fonc.2026.1863817

**Published:** 2026-06-02

**Authors:** Sevim Sahin, Abdullah Yesil

**Affiliations:** 1 Faculty of Engineering and Natural Sciences, Fenerbahce University, Department of Electrical and Electronics Engineering, Istanbul, Türkiye; 2 Department of Radiation Oncology, Medicana Bursa Hospital, Bursa, Türkiye

**Keywords:** AI-based segmentation, dosimetric comparison, hematopoietic stem cell transplantation, organ-at-risk sparing, total body irradiation, total marrow irradiation, treatment plan complexity, VMAT

## Abstract

**Background/objectives:**

Total body irradiation (TBI) is widely used in conditioning regimens prior to hematopoietic stem cell transplantation, but it is associated with significant radiation exposure to normal tissues. Total marrow irradiation (TMI) has emerged as a more targeted alternative, aiming to reduce organ-at-risk doses while maintaining target coverage. This study aimed to evaluate whether TMI can provide clinically meaningful organ sparing while maintaining acceptable treatment complexity using a paired dosimetric approach.

**Methods:**

Thirty adult patients with acute leukemia who previously received VMAT-based TBI were retrospectively included. For each patient, a corresponding VMAT-based TMI plan was generated using the same CT datasets. To ensure planning efficiency and standardization, target delineation for TMI was facilitated by AI-based auto-segmentation. Plan quality was assessed using the homogeneity index, and complexity was analyzed based on monitor units, segment number, and modulation factor. Paired comparisons were performed using the Wilcoxon signed-rank test.

**Results:**

TMI plans demonstrated a consistent and statistically significant reduction in dose to all evaluated organs at risk compared with TBI (p < 0.001). Mean doses to the heart, kidneys, and liver were reduced by approximately 5–6 Gy, and the lung dose was also significantly decreased. TMI provided improved dose homogeneity and showed significantly lower monitor units, segment number, and modulation factor compared with TBI.

**Conclusions:**

VMAT-based TMI reduced organ-at-risk doses while preserving target coverage and showing favorable plan quality and complexity metrics. AI-based auto-segmentation may reduce the workload of skeletal target delineation; however, treatment uncertainties related to patient positioning, respiratory motion, and field-junction dose matching should be carefully managed during clinical implementation.

## 1 Introduction

Total body irradiation (TBI) is a radiotherapy technique that delivers megavoltage photon beams to the entire body and represents an important component of conditioning regimens prior to hematopoietic stem cell transplantation (HSCT). The primary goals of TBI are to eradicate residual malignant cells that may persist after systemic chemotherapy, provide profound immunosuppression to prevent graft rejection, and ablate the patient’s native bone marrow to facilitate donor stem cell engraftment. Through these combined mechanisms, TBI contributes to improved transplantation success and disease control in various hematologic malignancies (1–3).

However, conventional TBI exposes the entire body to radiation and is therefore associated with substantial dose delivery to normal tissues. As a result, both acute and late toxicities may occur in critical organs, leading to complications such as interstitial pneumonitis in the lungs, radiation nephropathy in the kidneys, and hepatic sinusoidal obstruction syndrome. These toxicities remain a major limitation of conventional TBI approaches and have motivated the development of treatment strategies aimed at reducing unnecessary irradiation of normal tissues while maintaining adequate therapeutic effectiveness (4, 5).

Advances in modern radiotherapy technologies have enabled the development of more conformal irradiation techniques. Methods such as intensity-modulated radiation therapy (IMRT), volumetric modulated arc therapy (VMAT), and helical tomotherapy (HT) allow for improved dose shaping and more effective sparing of organs at risk compared with conventional TBI techniques (6–8). Building on these technological advances, more targeted irradiation strategies have been proposed. One such approach is total marrow irradiation (TMI), which selectively targets marrow-containing skeletal structures while reducing radiation exposure to surrounding normal tissues. By focusing radiation delivery on the bone marrow rather than the entire body, TMI aims to preserve the therapeutic benefits of TBI while potentially decreasing treatment-related toxicity (9).

Previous dosimetric studies have demonstrated that marrow-targeted irradiation techniques can substantially reduce doses to organs at risk, including the lungs, kidneys, liver, and brain, while maintaining acceptable target coverage (10–12). However, the specific clinical planning contribution of the present study lies in its paired design. To our knowledge, published data directly comparing VMAT-based TBI and VMAT-based TMI within the same patient cohort remain limited, particularly when plan complexity parameters are evaluated together with organ-at-risk dosimetry. Therefore, this study aimed to perform a paired dosimetric comparison between VMAT-based TBI and VMAT-based TMI treatment plans generated for the same patient cohort, with a particular focus on organ-at-risk doses, target dose characteristics, and treatment plan complexity.

## 2 Methods

### 2.1 Patient selection

This retrospective study included 30 adult patients (8 females and 22 males) diagnosed with acute myeloid leukemia (AML) or acute lymphoblastic leukemia (ALL) who underwent VMAT-based TBI prior to allogeneic stem cell transplantation at Medicana Bursa Hospital between 2020 and 2025. For the purpose of dosimetric comparison, additional VMAT-based TMI treatment plans were retrospectively generated on the same CT datasets for each patient. Patient characteristics are summarized in **Table 1**.

**Table 1:** Patient demographics.

Variable	Value
Number of Patients	30
Age (years), mean ± SD (range)	53.8 ± 12.6 (18–72)
Sex (Female/Male)	8/22
Diagnosis (AML/ALL)	13/17

Ethical approval for the study was obtained from the Non-Interventional Clinical Research Ethics Committee of Fenerbahçe University (Approval No: 93.2025fbu). Given the retrospective nature of the study, the requirement for informed consent was waived. All patient data were anonymized prior to analysis.

### 2.2 CT simulation, contouring and treatment planning

Patient CT scans were acquired using a GE Revolution Evo CT simulator with 70-cm bore (GE Healthcare, Chicago, IL, US). Patients were positioned in the head-first supine position with their arms alongside the body. The CT images were obtained with a slice thickness of 5 mm.

For TBI planning, the planning target volume (PTV) was generated by subtracting the total lung volume from the external body contour. The resulting PTV-TBI was trimmed to maintain a 3-mm distance from the lenses and cropped 3 mm beneath the skin surface. In our institutional VMAT-TBI protocol, the kidneys were not geometrically subtracted from the PTV-TBI to ensure continuous target coverage and avoid potential dose discontinuities (cold spots) within the mid-body. Instead, they were defined as separate organs at risk (OARs), and renal sparing was managed during inverse optimization by applying OAR dose constraints. Consequently, kidney dose parameters were evaluated and reported independently as OAR endpoints. For TMI planning, the target volume included the skeletal structures from the vertex to the proximal one-third of the femurs, including the mandible, ribs, vertebrae, and pelvis with a uniform 3-mm expansion. The distal femurs, lower legs, feet, and bones of the hands were not included in the PTV-TMI (13). Similarly, the PTV-TMI was kept 3 mm away from the lenses and cropped 3 mm inside the skin contour.

The original TBI treatments were contoured and planned using the Monaco treatment planning system (version 6.1.2.0, Elekta AB, Stockholm, Sweden) and delivered using a Versa HD (160 MLC) linear accelerator (Elekta AB, Stockholm, Sweden). The upper-body and lower-body plans were optimized separately. After the lower-body isocenter was positioned and the lower-body plan was completed, a plan sum was generated to evaluate the cumulative dose distribution. Particular attention was paid to the field junction between the upper- and lower-body plans. The matching of these fields was adjusted according to patient anatomy, particularly lower-extremity thickness. The final evaluation of this region was based on the summed dose distribution, with the aim of maintaining the dose in the junction region within approximately 10–14 Gy and avoiding clinically relevant cold or hot spots.

For the comparative TMI plans, the bone marrow target volume was delineated using the cloud-based MVision AI Contour+ software (MVision, Helsinki, Finland), as manual contouring of the entire skeletal system is highly time-consuming. Deep learning-based auto-segmentation was used to support planning efficiency, improve delineation consistency, and standardize the initial skeletal target generation. The AI-generated contours provided a structured starting point for TMI planning and were subsequently reviewed and refined by an expert physicist before plan optimization. This workflow was intended to reduce the manual contouring burden while maintaining expert oversight and clinically appropriate target definition.

**Figure 1:**
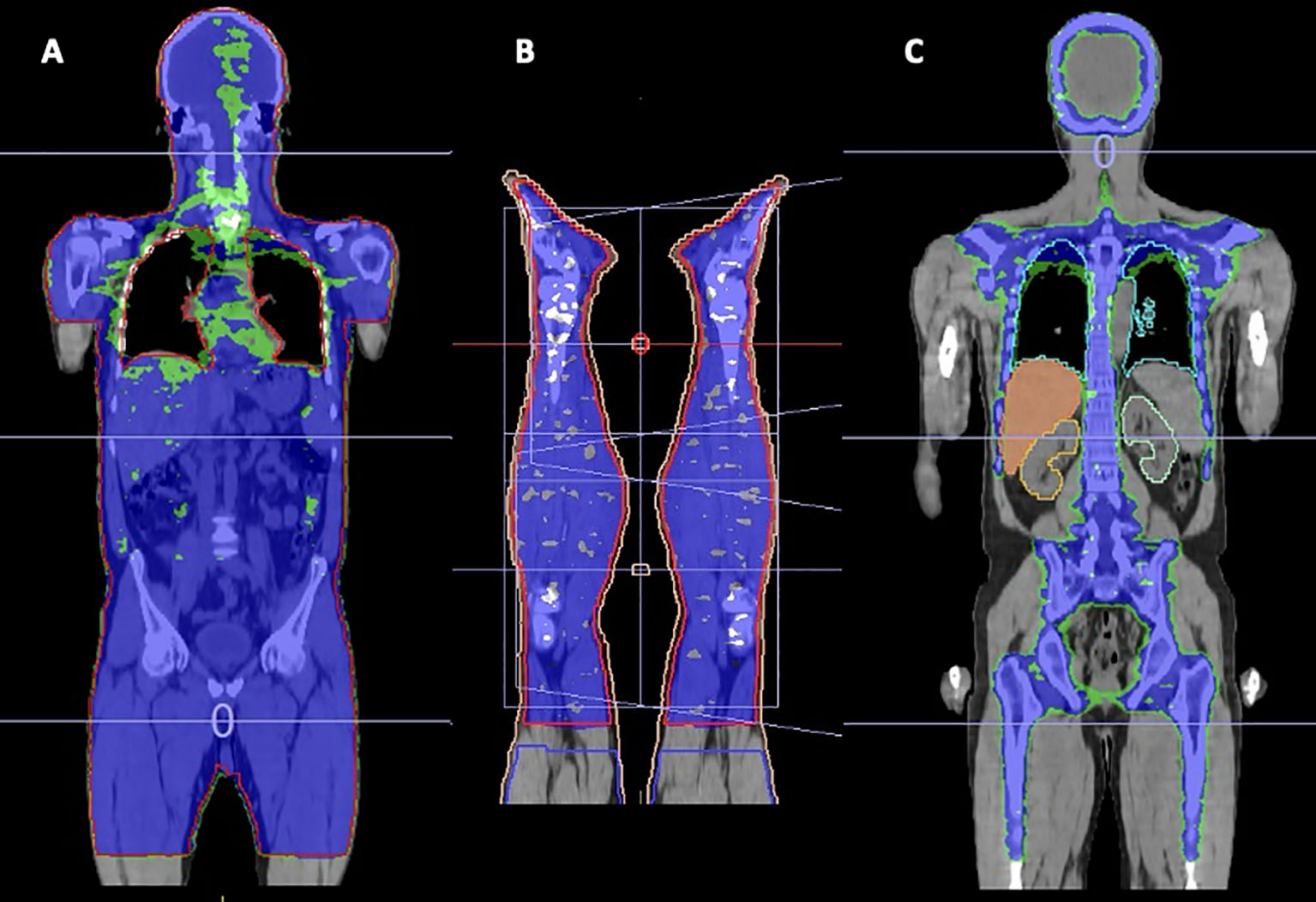
Representative coronal dose distributions for VMAT-based TBI and TMI plans. **(A)** Upper-body component of the VMAT-based TBI plan. **(B)** Lower-body component of the VMAT-based TBI plan, illustrating the additional lower-body treatment geometry. **(C)** Corresponding VMAT-based TMI plan, showing dose deposition mainly within the skeletal target. The displayed isodose lines correspond to 95% and 100% of the prescribed dose.

All patients received a total dose of 12 Gy delivered in six fractions of 2 Gy, administered twice daily, using 6-MV photon beams. For VMAT-based TBI planning, the upper-body component was planned using three isocenters. The first and third isocenters employed a single beam with two arcs, whereas the second isocenter utilized two beams, each consisting of two arcs. The lower-body component was planned with two additional isocenters. Therefore, the complete VMAT-based TBI plan consisted of five isocenters in total. For VMAT-based TMI planning, three isocenters were used with the same beam and arc arrangement described for the upper-body TBI component. Treatment plans were optimized to ensure that at least 90% of the PTV received 100% of the prescribed dose of 12 Gy. In addition, the maximum dose was constrained to remain below 15 Gy. Dose calculations were performed using the Monte Carlo algorithm implemented in the Monaco treatment planning system. Representative coronal dose distributions for the VMAT-based TBI upper-body component, TBI lower-body component, and VMAT-based TMI plan are shown in **Figure 1**.

### 2.3 Dosimetric evaluation

Treatment plans were evaluated using dose–volume histogram (DVH) parameters extracted from the Monaco treatment planning system. For target dose assessment, the homogeneity index (HI) was calculated as


$$HI=\frac{D2\%-D98\%}{D50\%}$$

Organs at risk (OARs) included the lenses, parotid glands, thyroid, brain, brainstem, esophagus, liver, lungs, heart, kidneys, and skin. For these structures, relevant dosimetric parameters such as mean dose and maximum dose to 0.03 cc were recorded where applicable. Specifically, the maximum dose to the left and right lenses, mean doses to the bilateral parotid glands, thyroid, brain, brainstem, esophagus, liver, lungs, heart, kidneys, and skin were evaluated. For selected serial organs, the maximum dose to 0.03 cc was additionally assessed.

In addition to dosimetric parameters, plan complexity metrics were also analyzed. These included the total number of monitor units (MU), segment number, and the modulation factor (MF), defined as MU per cGy.

### 2.4 Statistical analysis

Statistical analysis was performed using SPSS software (IBM SPSS Statistics, version 31.0.1.0, IBM Corp., Armonk, NY, USA). The distribution of the dosimetric parameters was assessed using the Shapiro–Wilk test. Since most paired variables did not follow a normal distribution, comparisons between VMAT-based TBI and TMI plans were performed using the Wilcoxon signed-rank test for paired samples. A p-value of less than 0.05 was considered statistically significant.

## 3 Results

### 3.1 Patient cohort and target coverage

**Figure 2:**
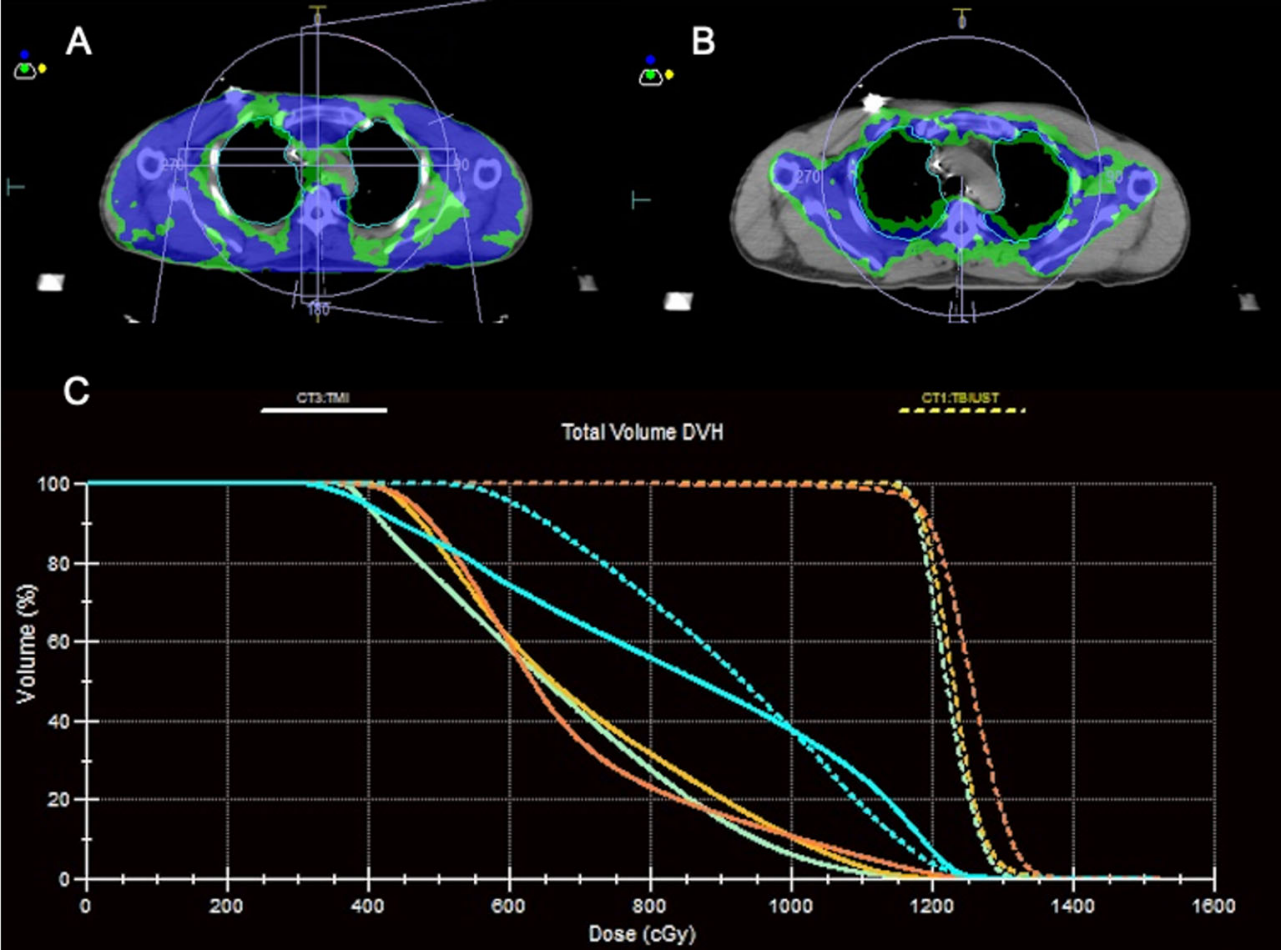
Representative axial dose distributions and DVH comparison of VMAT-based TMI and TBI plans. **(A)** Axial dose distribution from a representative TMI plan. **(B)** Axial dose distribution from the corresponding TBI plan at a comparable anatomical level. The displayed isodose lines correspond to 95% and 100% of the prescribed dose. **(C)** Total volume DVH curves for the TMI and TBI plans. Solid lines represent VMAT-based TMI plans, whereas dashed lines represent VMAT-based TBI plans. The color-coded DVH curves represent the left kidney (light green), right kidney (yellow), liver (orange), and total lung (cyan).

A total of 30 patients were included in the dosimetric comparison between VMAT-based TBI and TMI treatment plans. All treatment plans satisfied the predefined target coverage criterion, with at least 90% of the PTV receiving the prescribed dose of 12 Gy. This ensured that comparisons between TBI and TMI were performed under clinically acceptable and consistent planning conditions (**Figure 2**).

### 3.2 Major organs at risk

The comparison of major thoraco-abdominal organs is summarized in **Table 2**. TMI plans resulted in lower mean doses to all evaluated organs compared with TBI. The mean total lung dose decreased from 8.90 ± 0.51 Gy in TBI to 8.06 ± 0.48 Gy in TMI (p < 0.001).

**Table 2:** Paired comparison of mean doses to major thoraco-abdominal organs between TBI and TMI.

OAR	TBI (Mean ± SD)	TMI (Mean ± SD)	Mean difference (TBI-TMI) (Gy)	p-value
Mean Total Lung	8.90 ± 0.51	8.06 ± 0.48	0.84	<0.001
Mean Heart	12.34 ± 0.27	6.52 ± 0.74	5.82	<0.001
Mean Total Kidney	12.47 ± 0.16	6.31 ± 0.69	6.16	<0.001
Mean Liver	12.51 ± 0.16	6.92 ± 0.66	5.59	<0.001

**Figure 3:**
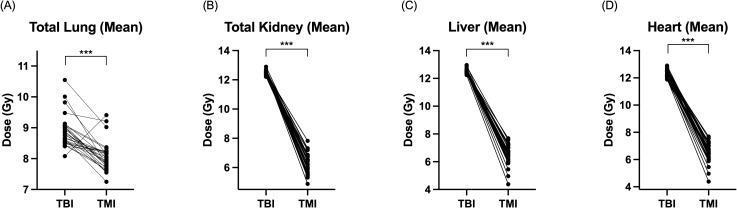
Paired comparison of mean doses to major thoraco-abdominal organs between VMAT-based TBI and TMI plans. **(A)** Mean total lung dose, **(B)** mean total kidney dose, **(C)** mean liver dose, and **(D)** mean heart dose. Each paired data point represents an individual patient. Significant dose reductions were observed with TMI for all evaluated organs (***p < 0.001).

Larger reductions were observed for the heart, kidneys, and liver. The mean heart dose decreased from 12.34 ± 0.27 Gy to 6.52 ± 0.74 Gy (p < 0.001), while the mean kidney dose decreased from 12.47 ± 0.16 Gy to 6.31 ± 0.69 Gy (p < 0.001). The mean liver dose was reduced from 12.51 ± 0.16 Gy in TBI to 6.92 ± 0.66 Gy in TMI (p < 0.001). Paired comparisons of these major organ doses are shown in **Figure 3**.

### 3.3 Secondary organs at risk

**Figure 4:**
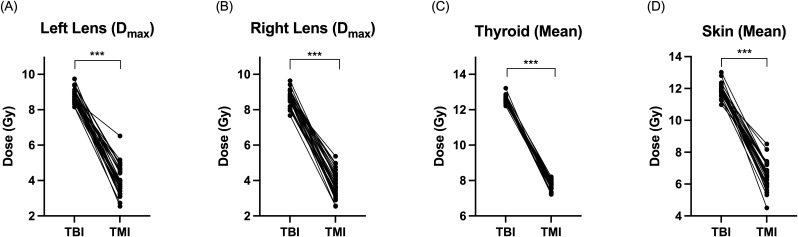
Paired comparison of doses to selected secondary organs at risk between VMAT-based TBI and TMI plans. **(A)** Left lens maximum dose (Dmax), **(B)** right lens maximum dose (Dmax), **(C)** mean thyroid dose, and **(D)** mean skin dose. Each paired data point represents an individual patient. Significant dose reductions were observed with TMI for all evaluated structures (***p < 0.001).

Dose comparisons for secondary organs at risk are summarized in **Table 3**. TMI plans showed significantly lower doses across most evaluated structures, including the lenses, parotid glands, thyroid, brain, brainstem, esophagus, heart maximum dose, and skin (all p < 0.001). The most pronounced reductions were observed for the lenses, thyroid, and skin, as illustrated in **Figure 4**. Specifically, the maximum doses to the left and right lenses decreased from approximately 8.7–8.8 Gy in TBI to 3.8–4.0 Gy in TMI. Similarly, the mean thyroid and skin doses were markedly reduced with TMI. A modest but statistically significant reduction was also observed in the maximum dose to the brain outside the target volume (brain–PTV D0.03cc), which decreased from 13.62 ± 0.38 Gy in TBI to 13.22 ± 0.65 Gy in TMI (p = 0.003).

**Table 3:** Comparison of doses to secondary organs at risk between TBI and TMI plans.

OAR	TBI (Mean ± SD)	TMI (Mean ± SD)	Mean difference (TBI-TMI) (Gy)	p-value
Left Lens Dmax	8.80 ± 0.39	3.99 ± 0.83	4.81	<0.001
Right Lens Dmax	8.67 ± 0.43	3.81 ± 0.71	4.86	<0.001
Left Parotid Mean (Gy)	12.60 ± 0.19	7.75 ± 0.31	4.86	<0.001
Right Parotid Mean (Gy)	12.63 ± 0.16	7.82 ± 0.30	4.81	<0.001
Thyroid Mean (Gy)	12.57 ± 0.18	7.77 ± 0.25	4.80	<0.001
Brain Mean (Gy)	12.58 ± 0.18	9.94 ± 0.59	2.64	<0.001
Brain − PTV D0.03cc (Gy)	13.62 ± 0.38	13.22 ± 0.65	0.39	0.003
Brainstem Mean (Gy)	12.44 ± 0.18	10.11 ± 0.64	2.34	<0.001
Brainstem D0.03cc (Gy)	13.53 ± 0.32	13.12 ± 0.66	0.42	<0.001
Esophagus Mean (Gy)	12.39 ± 0.29	8.70 ± 0.43	3.69	<0.001
Heart D0.03cc (Gy)	13.81 ± 0.26	12.33 ± 0.52	1.48	<0.001
Skin Mean (Gy)	11.90 ± 0.40	6.58 ± 0.83	5.32	<0.001

### 3.4 Plan quality and complexity

**Figure 5:**
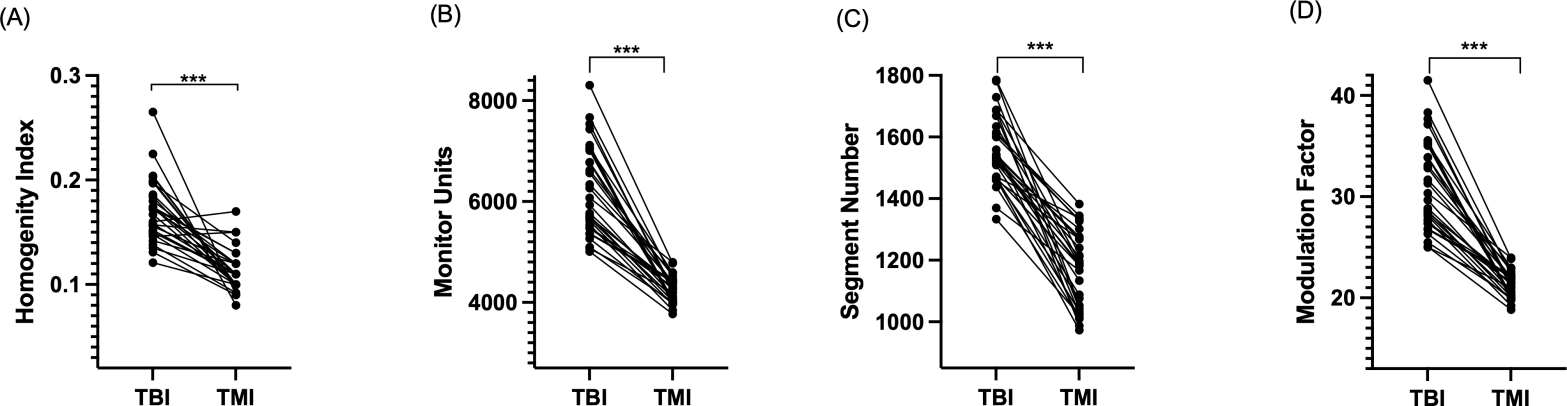
Paired comparison of plan quality and complexity metrics between VMAT-based TBI and TMI plans. **(A)** Homogeneity index, **(B)** monitor units, **(C)** segment number, and **(D)** modulation factor. Each paired data point represents an individual patient. Significant differences were observed for all evaluated plan quality and complexity metrics (***p < 0.001).

TMI plans showed significantly better dose homogeneity than TBI plans, as reflected by the lower homogeneity index (p < 0.001). Monitor units, segment number, and modulation factor were also significantly lower in TMI plans than in TBI plans (p < 0.001 for all). Plan quality and complexity metrics are summarized in **Table 4**. Paired comparisons of these plan quality and complexity metrics are presented in **Figure 5**.

**Table 4:** Comparison of plan quality and complexity metrics between TBI and TMI plans.

Parameter	TBI (Mean ± SD)	TMI (Mean ± SD)	p-value
Homogeneity Index	0.171 ± 0.030	0.113 ± 0.020	<0.001
Monitor Units	6192.8 ± 935.4	4254.3 ± 264.4	<0.001
Segment Number	1557 ± 112.7	1169.0 ± 123.5	<0.001
Modulation Factor (MU/cGy)^1^	30.96 ± 4.68	21.27 ± 1.32	<0.001

^1^ MF has been calculated per fraction (2 Gy).

## 4 Discussion

The main limitation of conventional TBI is the unavoidable irradiation of normal tissues, which has long been associated with both acute and late toxicities. Among these, pulmonary toxicity remains one of the most clinically important concerns, as pneumonitis has consistently been described as a major dose-limiting complication (4, 14, 15). In addition to lung injury, renal, ocular and other toxicities have also been reported after TBI, particularly when higher doses are used or long-term survivors are evaluated (16–19). These concerns have been a major reason for developing more selective conditioning approaches such as TMI. Beyond simply reducing side effects, this shift is driven by a critical therapeutic trade-off; while escalating the radiation dose is a proven strategy for lowering relapse rates, its clinical use has long been hindered by the heavy toll of conventional TBI on healthy organs (20–22). In this context, TMI has been proposed as a more selective irradiation approach that may improve organ-at-risk sparing while maintaining target coverage. However, this potential advantage should be interpreted together with the technical complexity and clinical implementation requirements of marrow-targeted irradiation. Our findings are consistent with this rationale. In the present paired dosimetric analysis, VMAT-based TMI reduced dose to all major thoraco-abdominal organs evaluated, with particularly marked reductions in the lungs, kidneys, heart, and liver. A similar pattern was observed for several secondary organs at risk, including the lenses, parotid glands, thyroid, brain, brainstem, esophagus, and skin. Taken together, these results suggest that the dosimetric advantage of TMI is not limited to a single anatomical region but extends across multiple organs that are commonly implicated in TBI-related toxicity.

An important strength of the present study is the paired planning design, which enabled a direct comparison between two VMAT-based approaches with different target definitions in the same patient cohort, while minimizing the effect of anatomical variability. Since all plans met the same minimum target coverage requirement, the comparison was performed under clinically consistent conditions, and the observed reductions in organ-at-risk doses were achieved without compromising basic target coverage. In addition, the inclusion of plan complexity metrics, including monitor units, segment number, modulation factor, and homogeneity index, provides further insight into the practical deliverability of TMI in a clinical setting. These aspects strengthen the planning relevance of our findings by showing that organ-at-risk dose reductions were achieved together with favorable plan quality and complexity metrics in this cohort.

This organ-sparing pattern is also in line with previous studies comparing marrow-targeted irradiation with conventional TBI. Köksal et al., in a paired planning study using helical tomotherapy, reported significant reductions in organ-at-risk doses while maintaining target coverage (23). In addition, Aydogan et al. showed that VMAT-based TMI can maintain target coverage while reducing organ-at-risk doses and supporting efficient treatment delivery compared to other TMI techniques (24). Similarly, early feasibility studies have demonstrated that VMAT-based TMI can achieve effective target coverage while enabling meaningful sparing of normal tissues, with acceptable delivery accuracy confirmed by quality assurance analyses (25).

Although the present study focuses on the dosimetric comparison between VMAT-based TBI and TMI, TBI remains an established standard conditioning approach before allo-HSCT. Recent clinical evidence also supports its continued role by demonstrating associations between TBI dose and transplantation outcomes, including relapse, non-relapse mortality, toxicity, overall survival, and progression-free survival (26). Historical TBI series have documented pulmonary complications, ocular injury including cataract formation, renal dysfunction, and hepatic toxicity, whereas TMI studies and recent reviews have generally reported favorable organ sparing with the potential for improved tolerability (27, 28). At the same time, toxicity is not completely eliminated, particularly at higher dose levels. This point is important because it places the benefit of TMI in a realistic context: marrow-targeted irradiation appears to reduce normal tissue exposure and may improve tolerability, but it does not remove the need for careful dose selection and clinical monitoring (29).

The reduction in lung dose observed in our study is particularly relevant. Since pulmonary toxicity has traditionally been one of the principal barriers in TBI-based conditioning, any meaningful decrease in lung dose may have potential clinical relevance. Similar considerations apply to the kidneys and lenses. Renal toxicity has been associated with TBI dose-related effects in previous studies, and ocular exposure remains a well-recognized source of late complications. In this context, the lower kidney and lens doses achieved with TMI strengthen the dosimetric relevance of our dosimetric findings. However, one patient-level exception was observed, in which the lung dose was slightly higher with TMI than with TBI. This finding was most likely related to patient-specific thoracic anatomy and the spatial proximity between the ribs, which are included in the TMI target, and the adjacent lung volume. Therefore, although TMI provided significant lung dose reduction at the group level, individual anatomical variations may influence the degree of lung sparing in selected patients.

An additional important observation was that the improved organ sparing achieved with TMI did not appear to be associated with a major increase in measured plan complexity. TMI plans showed a significantly lower homogeneity index, monitor units, segment number, and modulation factor compared with TBI plans. These findings indicate that, in this paired planning comparison, the dosimetric gains of TMI were achieved with lower overall plan complexity metrics rather than at the cost of increased measured plan complexity.

From a practical perspective, one of the main challenges of TMI is the increased workload associated with contouring, particularly for the extensive skeletal target volume. As previously noted by Köksal et al., this process can be time-consuming and may limit the feasibility of TMI in high-volume clinical settings (23). In addition to contouring workload, the clinical implementation of TMI requires careful patient selection, robust immobilization, image-guided setup verification, multi-isocenter planning expertise, and patient-specific quality assurance. These requirements are particularly important because the skeletal target extends over a large anatomical region, includes anatomically complex structures such as the ribs, and may be affected by respiratory motion and field-junction uncertainties. Although TMI showed favorable dosimetric and plan complexity results in the present paired planning comparison, its routine implementation may be constrained by these practical considerations. However, recent advances in automated segmentation, particularly AI-based contouring tools, have the potential to reduce part of the contouring workload. With improved efficiency and consistency in target delineation, such technologies may facilitate the broader clinical adoption of TMI in the future, provided that careful expert review, planning validation, and treatment-specific quality assurance are maintained.

This study has several limitations. First, it is a retrospective planning comparison and does not include direct clinical toxicity or survival data. Therefore, the reduction in organ doses observed with TMI should be interpreted as a potential rather than proven clinical advantage. Second, long-term late-effect data for TMI remain more limited than the much longer clinical experience available for conventional TBI. Finally, the present analysis reflects the planning conditions of a single institution. Nevertheless, to our knowledge, direct paired comparisons of VMAT-based TBI and VMAT-based TMI within the same patient cohort remain limited, particularly when organ-at-risk sparing and plan complexity parameters are evaluated together. In this context, the present study may provide useful additional evidence on the practical differences between these two approaches.

Although the original TBI treatments were delivered with routine IGRT-based setup verification according to the institutional workflow, the present study was based on planned dose distributions calculated on simulation CT datasets for both techniques. Therefore, respiratory motion, fraction-specific setup variation, and delivered-dose accumulation were not evaluated. However, recent literature highlights that these factors are critical for delivered-dose accuracy and the clinical implementation of multi-isocenter VMAT-based TBI (30, 31), and they should be addressed in future delivered-dose studies.

Overall, the present study shows that VMAT-based TMI can substantially reduce dose to multiple organs at risk while preserving target coverage and showing favorable plan quality and complexity metrics compared with VMAT-based TBI. Given that toxicity has always been one of the principal limitations of conventional TBI, these findings support TMI as a more selective conditioning approach with the potential to improve normal tissue sparing.

## 5 Conclusions

In this study, VMAT-based TBI and TMI plans were directly compared using a paired planning approach in the same patient cohort, enabling a controlled evaluation of dosimetric differences under consistent planning conditions. While the organ-sparing advantage of TMI has been previously reported, our findings confirm that these reductions can be achieved within the same treatment platform and patient anatomy. Importantly, this dosimetric benefit was not associated with a substantial increase in overall plan complexity. These results suggest that VMAT-based TMI may provide a clinically practical approach for improving organ-at-risk sparing while maintaining feasible treatment delivery. In addition, the integration of AI-based automated segmentation tools into clinical workflows may facilitate the broader implementation of this approach. However, the impact of these advantages on long-term clinical outcomes remains to be established.

## Data Availability

The raw data supporting the conclusions of this article will be made available by the authors, without undue reservation.
